# The Role of Interleukin-33 in Head and Neck Squamous Cell Carcinoma Is Determined by Its Cellular Sources in the Tumor Microenvironment

**DOI:** 10.3389/fonc.2020.588454

**Published:** 2021-02-09

**Authors:** Liang Peng, Wei Sun, Lin Chen, Wei-Ping Wen

**Affiliations:** ^1^ Department of Otolaryngology, The First Affiliated Hospital, Sun Yat-sen University, Guangzhou, China; ^2^ Otorhinolaryngology Institute, Sun Yat-sen University, Guangzhou, China; ^3^ Department of Otolaryngology, The Sixth Affiliated Hospital, Sun Yat-sen University, Guangzhou, China

**Keywords:** IL-33, alarmin, head and neck squamous cell carcinoma, tumor biomarker, tumor microenvironment

## Abstract

**Objectives:**

To investigate the role of interleukin-33 (IL-33) in head and neck squamous cell carcinoma (HNSCC).

**Materials and Methods:**

RNA-seq data of 520 cases of HNSCC were retrieved from The Cancer Genome Atlas. The tumor microenvironment was deconstructed by xCell using bulk RNA-seq data. The cohort was dichotomized by the median IL-33 expression level. Immune cell components and molecular markers were compared between the high and low IL-33 groups. The prognostic value of IL-33 was evaluated by the log-rank test. Differential gene expression analysis and KEGG pathway enrichment analysis were also conducted. The relationship between the IL-33 expression level and the abundance of its potential cellular sources was evaluated by Pearson’s partial correlation test. Subgroup analysis was conducted in laryngeal, oropharyngeal, and oral cavity squamous cell carcinoma (LSCC, OPSCC, and OCSCC).

**Results:**

The role of IL-33 in HNSCC was heterogeneous among tumors at different sites. In LSCC, IL-33 may increase the extent of malignancy of tumor cells and act as a pro-tumor factor. In OCSCC, IL-33 may play a role in orchestrating the immune responses against tumor cells and act as an antitumor factor. The role of IL-33 in OPSCC was undetermined. IL-33 in LSCC was mainly derived from endothelial cells, while IL-33 in OCSCC was mainly derived from endothelial and epithelial cells.

**Conclusion:**

According to the different sources of IL-33 in LSCC and OCSCC, we propose a hypothesis that stroma-derived IL-33 could favor tumor progression, while epithelial-derived IL-33 could favor antitumor immune responses in HNSCC.

## Introduction

Head and neck squamous cell carcinoma (HNSCC) is a heterogeneous disease that originates in the epithelial cells of the mucosal linings of the upper aerodigestive tract (oral cavity, oropharynx, hypopharynx, or larynx) (1). Tobacco and alcohol use or infection with human papillomavirus (HPV), are thought to be associated with the incidence of HNSCC (2, 3). Worldwide, more than 800,000 individuals were diagnosed with HNSCC, and more than 400,000 patients died from it in 2018 (4). Although the treatment modalities of HNSCC are constantly improving, the treatment effects for advanced disease are still unsatisfactory (5). During the last decade, immunotherapy, especially the clinical application of immune checkpoint inhibitors, has shown promising results in the war against malignancy and has shed some light on treatments for HNSCC (6).

Interleukin-33 (IL-33), a member of the IL-1 family, is constitutively expressed at high levels in the nuclei of various cell types, including endothelial, epithelial and fibroblast-like cells (7). IL-33 is released by necrotic or damaged cells and secreted into the extracellular space, where it can bind to a heterodimer formed by its specific primary receptor IL-1 receptor-like 1 and co-receptor, IL-1 receptor accessory protein, acting as an alarmin (8, 9). IL-33 was first described as an inducer of type 2 immune responses that activates T helper 2 (T_H_2) cells and mast cells (8). With accumulated evidence, IL-33 has also been proven to stimulate many other immune cells, including group 2 innate lymphoid cells (ILC2s) (10), regulatory T (T_reg_) cells (11), T helper 1 (T_H_1) cells (12), CD8^+^ T cells (13), natural killer (NK) cells (14), dendritic cells (DCs) (15), eosinophils, basophils (16) and macrophages (17). Due to its pleiotropic effects, IL-33 plays an important role in tissue homeostasis, infection, inflammation and cancer (7).

However, the role of IL-33 in cancer remains controversial, with pro-tumor and antitumor effects in different settings (18–20). Intratumor IL-33 can directly act on cancer cells or indirectly act on the tumor microenvironment (TME) (20). In HNSCC, several studies have demonstrated that IL-33 may favor tumor progression by promoting tumor aggressiveness, angiogenesis or modulation of the tumor immune microenvironment (TIME) (21–24). The role of IL-33 in HNSCC is still undetermined.

In the current study, we aimed to explore the role of IL-33 in HNSCC using mRNA-seq data from The Cancer Genome Atlas (TCGA) and to better understand its potential as a biomarker or therapeutic target in HNSCC.

## Method and Materials

### Data Set

A total of 520 HNSCC cases from the TCGA were included in this study. The mRNA-seq, genetic mutation and copy-number alteration (CNA) data of primary tumors and the clinical information of patients was obtained from the cBioPortal for Cancer Genomics in February 2020 (http://www.cbioportal.org) (25, 26). Gene expression values were presented as RNA-Seq by Expectation Maximization (RSEM) data normalized to the upper quartile of total reads within each sample (27). Immune cytolytic activity (CYT), which can reflect antitumor immune responses, was calculated as the geometric mean of the mRNA expression of granzyme A and perforin in RSEM, as previously reported (28).

### TME Decomposition

The bulk RNA-seq data of tumor samples were used to dissect the TME using xCell (29). Enrichment scores for 64 cell types, including immune cells, stromal cells and epithelial cells, were calculated to represent the abundance of each cell component within the tumor.

### Statistical Analysis

The expression level of IL-33 was compared among different subgroups defined by clinicopathological characteristics using the Mann-Whitney U test or Kruskal-Wallis test. Patients were divided into two groups based on the median IL-33 expression level. The overall survival (OS) was compared between the high IL-33 group and the low IL-33 group using the log-rank test. To evaluate the effects of IL-33 on tumor immune compositions, xCell scores of 20 predefined types of immune cells were compared between the high IL-33 group and the low IL-33 group using the Mann-Whitney U test, and the Benjamini-Hochberg method was used for multiple testing correction (30). The correlations between the IL-33 expression level and the abundance of the main sources of IL-33 (epithelial cells, endothelial cells, pericytes, fibroblasts, and smooth muscle cells represented by xCell scores) were evaluated by the Pearson partial correlation test. Differential gene expression analysis between the high IL-33 group versus the low IL-33 group was performed with the cBioPortal platform. KEGG pathway enrichment analysis of differentially expressed genes was performed with the DAVID platform (version 6.8, https://david.ncifcrf.gov) (31, 32). SPSS version 22.0 (IBM Corporation, Armonk, NY, USA) was used for statistical analysis. Two-tailed p-values < 0.05 were considered statistically significant.

## Results

### IL-33 Expression in Subgroups

**Figure 1:**
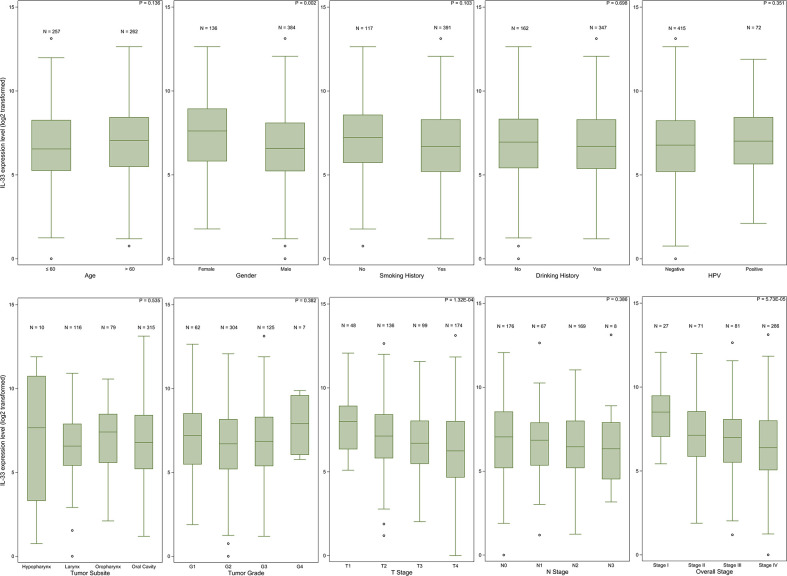
The IL-33 expression level in clinicopathological subgroups. Boxplots represent values within the interquartile range (IQR) (boxes) and 1.5 × IQR (whiskers). Outliers are plotted as values > 1.5 × IQR (circles). P-values were calculated by the Mann-Whitney U test or Kruskal-Wallis test.

The expression level of IL-33 in tumor samples from female patients was higher than that in tumor samples from male patients (p = 0.002). Patient age, smoking history or drinking history did not influence the expression level of IL-33. The earlier the pathologic T stage (American Joint Committee on Cancer [AJCC] staging system) of the tumor was, the higher the expression level of IL-33 was. However, the pathologic N stage (AJCC staging system) of the tumor did not influence the expression level of IL-33. In addition, we did not find any relationship between the expression level of IL-33 and the tumor site, tumor grade or HPV infection status (**Figure 1**, **Supplementary Table S1**).

### IL-33 and TIME

**Figure 2:**
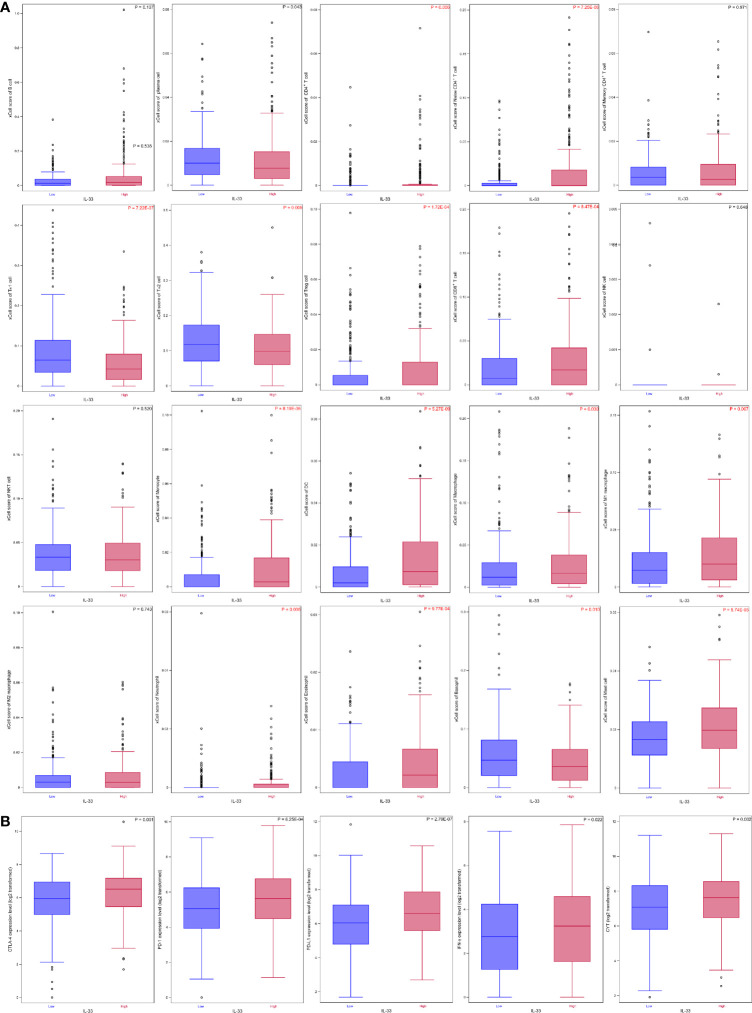
The IL-33 expression level and tumour immune microenvironment in HNSCC. **(A)** IL-33 expression level and immune cell abundances represented by xCell scores; **(B)** IL-33 expression level and molecular markers for tumour immunity. Boxplots represent values within the interquartile range (IQR) (boxes) and 1.5 × IQR (whiskers). Outliers are plotted as values > 1.5 × IQR (circles). P-values were calculated by the Mann-Whitney U test. The Benjamini-Hochberg method was used for multiple testing correction in **(A)**, and p-values with false discovery rates < 0.05 are shown in red. CYT, cytolytic activity.

The abundance of 20 types of immune cells, including B cells, plasma cells, CD4^+^ T cells, naïve CD4^+^ T cells, memory CD4^+^ T cells, T_H_1 cells, T_H_2 cells, T_reg_ cells, CD8^+^ T cells, NK cells, natural killer T (NKT) cells, monocytes, DCs, macrophages, M1 macrophages, M2 macrophages, neutrophils, eosinophils, basophils, and mast cells, in the tumor samples in the high IL-33 group and the low IL-33 group were compared. We found that 3 types of immune cells (T_H_1 cells, T_H_2 cells, and basophils) accumulated more in low IL-33 tumors than in high IL-33 tumors, while 11 types of immune cells (CD4^+^ T cells, naïve CD4^+^ T cells, T_reg_ cells, CD8^+^ T cells, monocytes, DCs, macrophages, M1 macrophages, neutrophils, eosinophils, and mast cells) accumulated more in high IL-33 tumors than low IL-33 tumors after multiple testing correction (**Figure 2A**, **Supplementary Table S2**). Several important molecular markers for tumor immunity were also compared. We found that interferon-γ (IFN-γ) and CYT, which reflect the activity of the antitumor immune response, were higher in high IL-33 tumor samples. The immunoregulatory molecules cytotoxic T-lymphocyte antigen 4 (CTLA-4), programmed death receptor 1 (PD-1), and programmed death ligand 1 (PD-L1) were also higher in high IL-33 tumor samples than in low IL-33 tumor samples (**Figure 2B**, **Supplementary Table S2**).

### Prognostic Value of IL-33

**Figure 3:**
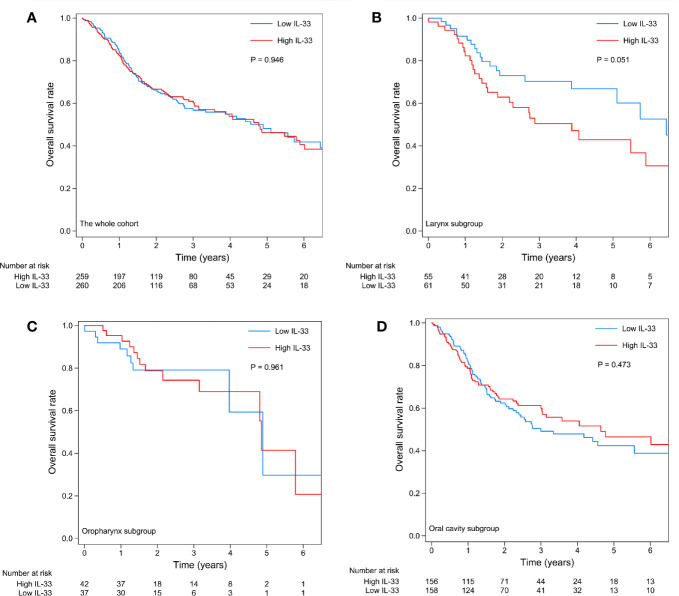
Kaplan-Meier curves of the high IL-33 and low IL-33 groups in the whole cohort **(A)** and in the laryngeal squamous cell carcinoma **(B)**, oropharyngeal squamous cell carcinoma **(C)**, and oral cavity squamous cell carcinoma **(D)** groups. P-values were calculated by the log-rank test.

A total of 519 cases with follow-up data were included in the survival analysis. The expression level of IL-33 had no prognostic value in the whole cohort (**Figure 3A**). Considering the heterogeneity among HNSCCs originating in different sites, we further investigated the prognostic value of IL-33 in subgroups of tumors of the larynx, oropharynx, and oral cavity (the hypopharynx subgroup was excluded due to the small number of cases). In patients with laryngeal squamous cell carcinoma (LSCC), high expression of IL-33 in tumor samples was an unfavorable prognostic factor, with a strong tendency towards statistical significance (p = 0.051) (**Figure 3B**). If we adjusted tumor stage (stage III/IV vs. stage I/II) in the survival analysis, the p-value of the log-rank test became 0.020, indicating statistical significance (data not shown). In patients with oropharyngeal squamous cell carcinoma (OPSCC), the expression level of IL-33 had no prognostic value (**Figure 3C**). In patients with oral cavity squamous cell carcinoma (OCSCC), the high expression of IL-33 in tumor samples seemed to be associated with favorable OS, as shown by the survival curves (**Figure 3D**), although without statistical significance (p = 0.473). Obviously, the prognostic value of IL-33 for HNSCCs was heterogeneous among tumors from different sites.

### Heterogeneity of IL-33’s Effects on TIME and Signaling Pathways

We compared the TIME between high IL-33 and low IL-33 samples separately in LSCC, OPSCC, and OCSCC. In LSCC, only the abundance of T_H_1 cells was found to be significantly different, which was higher in the low IL-33 group than in the high IL-33 group (**Supplementary Figure S1A**). No difference in molecular markers was detected (**Supplementary Figure S1B**). In OPSCC, although five types of immune cells (B cells, naïve CD4^+^ T cells, memory CD4^+^ T cells, DCs, and mast cells) were more abundant in the high IL-33 group (p < 0.05), none of them remained statistically significant after multiple testing correction (**Supplementary Figure S2A**). Regarding the molecular markers, PD-L1 and IFN-γ were more highly expressed in the high IL-33 group (**Supplementary Figure S2B**). In OCSCC, 10 types of immune cells (naïve CD4^+^ T cells, T_reg_ cells, CD8^+^ T cells, monocytes, DCs, macrophages, M1 macrophages, neutrophils, eosinophils, and mast cells) were more abundant in the high IL-33 group, while 4 types of immune cells (plasma cells, T_H_1 cells, T_H_2 cells, and basophils) were more abundant in the low IL-33 group (**Supplementary Figure S3A**). Regarding the molecular markers, CTLA-4, PD-1, PD-L1, IFN-γ, and CYT were all higher in the high IL-33 group than in the low IL-33 group (**Supplementary Figure S3B**).

**Figure 4:**
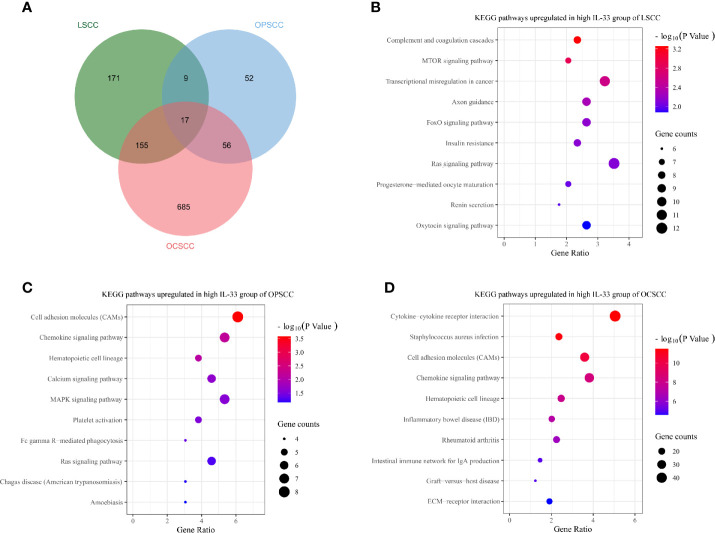
**(A)** Venn diagram depicting genes expressed at higher levels in the high IL-33 groups of LSCC, OPSCC, and OCSCC. Bubble plots depicting upregulated KEGG pathways in the high IL-33 groups of LSCC **(B)**, OPSCC **(C)**, and OCSCC **(D)**. KEGG pathway enrichment analysis was conducted with the DAVID platform. LSCC, laryngeal squamous cell carcinoma; OPSCC, oropharyngeal squamous cell carcinoma; OCSCC, oral cavity squamous cell carcinoma.

Genes differentially expressed between the high and low IL-33 groups were extracted on the cBioPortal platform with criteria of log ratio ≥ 0.5 and q-value < 0.05. Excluding IL-33, we found that 352, 134, and 913 genes were expressed at higher levels in the high IL-33 group than in the low IL-33 group in the LSCC, OPSCC, and OCSCC data sets, respectively (**Figure 4A**). These differentially expressed genes were subjected to KEGG pathway enrichment analysis with the DAVID platform. We plotted the 10 enriched pathways with the lowest p-values (**Figures 4B–D**). In LSCC, pathways related to cell survival, cell cycle, cell proliferation, and tumorigenesis were enriched in the high IL-33 group (**Figure 4B**). In OPSCC, pathways related to immune response, inflammation, cell survival, and cell proliferation were enriched in the high IL-33 group (**Figure 4C**). In OCSCC, pathways related to the immune response and inflammation were enriched in the high IL-33 group (**Figure 4D**).

### Possible Reasons for the Heterogeneity

**Figure 5:**
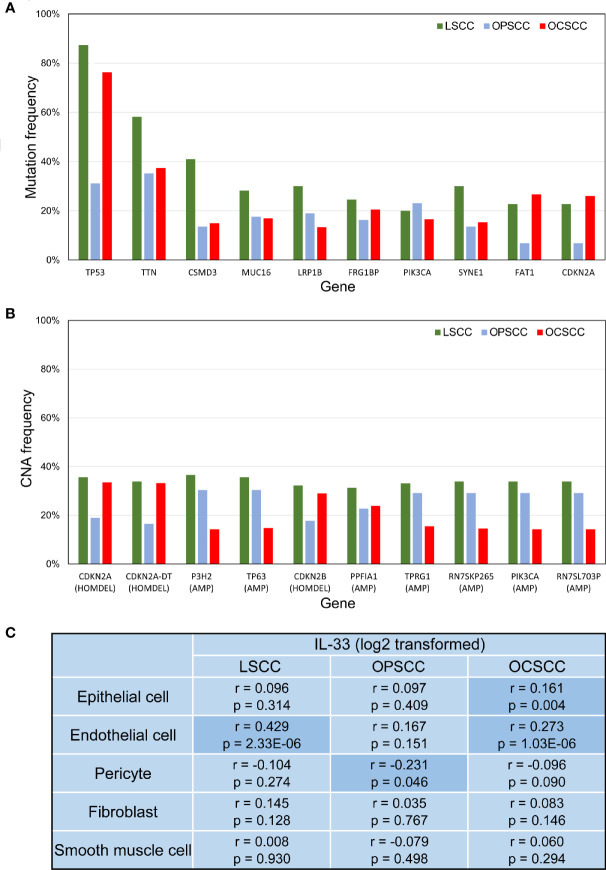
**(A)** Bar plot depicting the mutation frequencies of 10 genes with the highest average frequencies. **(B)** Bar plot depicting CNA frequencies of the 10 genes with the highest average frequencies. **(C)** Correlation between IL-33 expression level and the abundance of potential cellular sources. P-values were calculated by Pearson’s partial correlation test; r indicates the correlation coefficient. LSCC, laryngeal squamous cell carcinoma; OPSCC, oropharyngeal squamous cell carcinoma; OCSCC, oral cavity squamous cell carcinoma; CNA, copy-number alteration; HOMDEL, homozygous deletion; AMP, amplification.

Although LSCC, OPSCC, and OCSCC could be classified as HNSCC, the somatic genetic changes were not identical among these different types of tumors (**Figures 5A–B**). This may partly explain the heterogeneity of IL-33’s effects among these tumors. Considering that the heterogeneity of IL-33’s effects may be due to the different cellular sources of IL-33 in the TME (33), we tried to determine the potential sources of IL-33. In the TME, the potential sources of IL-33 were epithelial cells (including normal epithelial cells and tumor cells), endothelial cells, pericytes, fibroblasts, and smooth muscle cells. We investigated the correlation between the expression level of IL-33 and the abundance of one potential source while controlling the other potential sources. In LSCC, the expression level of IL-33 was positively correlated with the abundance of endothelial cells, indicating that endothelial cells may be the main source of IL-33 (**Figure 5C**). In OPSCC, the expression level of IL-33 was negatively correlated with the abundance of pericytes (**Figure 5C**). There was no indication that IL-33 was derived from one of these potential sources in OPSCC. A study reported that IL-33 could be expressed in B cells (34). Considering the wealth of lymphoid tissue around the oropharynx and the positive correlation between the expression level of IL-33 and the abundance of B cells suggested by **Supplementary Figure S2A**, it is suggested that IL-33 in OPSCC may be from B cells. In OCSCC, the expression level of IL-33 was positively correlated with the abundance of endothelial cells and epithelial cells, indicating that endothelial cells and epithelial cells may be the main sources of IL-33 (**Figure 5C**).

## Discussion

In the current study, we investigated the role of IL-33 in HNSCC using TCGA data sets. As shown in **Figure 2**, IL-33 could modulate the TIME of HNSCC, which is in accordance with previous studies (18–20). However, no prognostic effect of IL-33 was found in HNSCC. Considering that HNSCC is a group of heterogeneous diseases, we further conducted subgroup analyses in LSCC, OPSCC, and OCSCC. We found that the prognostic effects of IL-33 were heterogeneous among these tumors.

In LSCC, a high expression level of IL-33 in tumors was associated with poor OS. The TIME of LSCC was not correlated with the expression level of IL-33, except that high IL-33 was associated with a lower abundance of T_H_1 cells. The KEGG pathway enrichment analysis revealed that high IL-33 may increase the extent of malignancy of tumor cells in LSCC, which could explain the negative prognostic role of IL-33. In addition, we found that the angiolymphatic invasion rate of the high IL-33 group was much higher than that of the low IL-33 group in LSCC (23/34 vs. 13/44; chi-square test, p = 0.002). In contrast, the role of IL-33 in OCSCC seemed to be distinct. We found that immune cell components in the OCSCC TME were significantly altered by IL-33. In OCSCC with high IL-33, more naïve CD4^+^ T cells, CD8^+^ T cells, DCs, and M1 macrophages were recruited, which could favor antitumor responses. Interestingly, T_reg_ cells were also upregulated in the IL-33 high group, probably in response to the accumulation of cytolytic immune cells to maintain immune homeostasis (35). Notably, both antitumor T_H_1 and pro-tumor T_H_2 cells were downregulated in the high IL-33 group. As for the other altered myeloid cells, their roles in tumors are still ambiguous and need to be investigated further (36). IL-33 also modulated OCSCC TIME by increasing IFN-γ and CYT, which were both related to active antitumor responses. Concurrent with the upregulation of IFN-γ and CYT, immune checkpoint molecules—PD-1, PD-L1 and CTLA-4 were also upregulated, indicating that the high IL-33 group of OCSCC may respond well to immune checkpoint inhibitors. The KEGG pathway enrichment analysis also supported the role of IL-33 in the modulation of the TIME. Overall, a high expression level of IL-33 was associated with antitumor immune responses in OCSCC, which may explain the insignificantly favorable prognosis of the high IL-33 group. In OPSCC, IL-33 had no prognostic value. Whether IL-33 has a role in the modulation of the TIME or the progression of tumors in OPSCC is still unknown.

**Figure 6:**
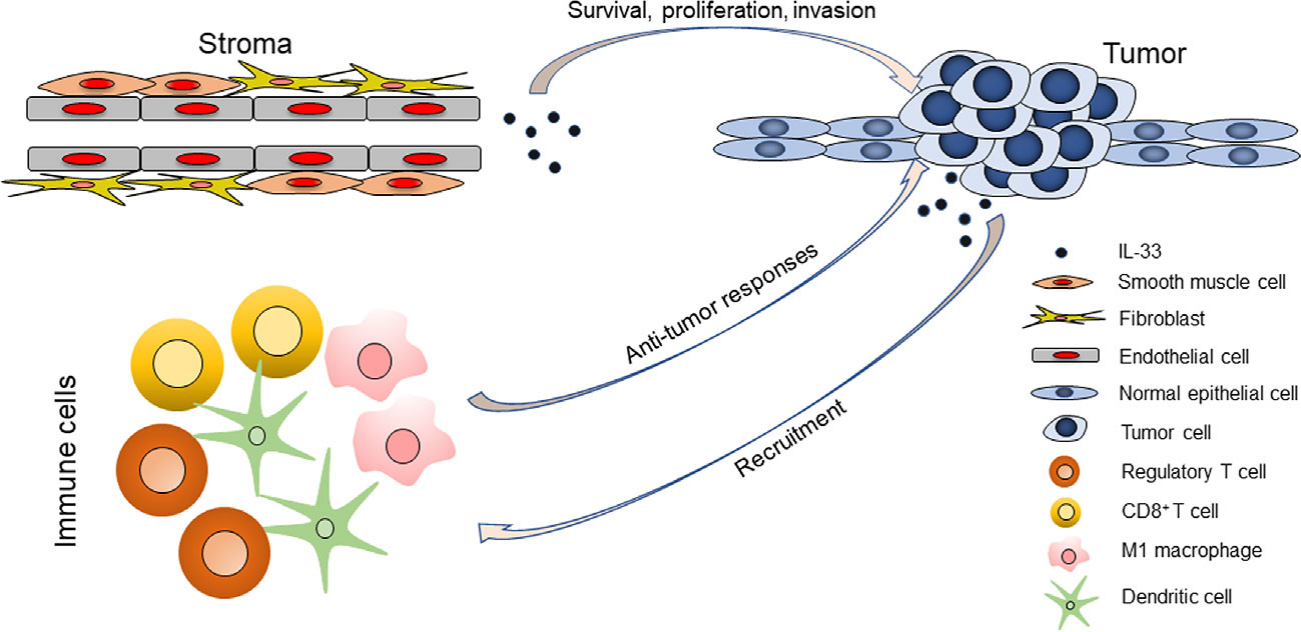
The roles of IL-33 in head and neck squamous cell carcinoma are determined by its cellular sources.

We propose a hypothesis that the cellular sources of IL-33 in the TME may determine its distinct roles in HNSCC (**Figure 6**). IL-33 from the stroma (particularly endothelial cells) could support the survival and proliferation of tumor cells and even directly or indirectly contribute to the invasiveness of tumor cells, but it is unlikely that this occurs through modulation of the TIME. IL-33 from the stroma could be a pro-tumor factor, as it is in the setting of LSCC. IL-33 from epithelial cells (normal and cancerous cells) could act as an alarmin and orchestrate antitumor immune responses. In the setting of OCSCC, both stroma- and epithelium-derived IL-33 exists and has contradictory roles, which may explain the statistical insignificance of the prognostic value of IL-33.

Several limitations should be noted when interpreting this study. First, the mRNA expression level rather than the protein expression level of IL-33 was analyzed, and we could not ascertain the levels of different forms of IL-33 or the functioning sites (whether nucleus or extracellular space). Second, compared to the single-cell technique, the accuracy of determining TME composition from bulk RNA-seq data is inevitably undermined by the limitations of the algorithm. Third, this is a cross-sectional study, and only correlation could be deduced. The causal relationship between IL-33 and others should be regarded as hypothetical and validated by well-designed *in vitro/vivo* experiments.

In conclusion, we found that IL-33 has a heterogeneous role in HNSCC, which is probably determined by the cellular sources of IL-33. Specifically, stroma-derived IL-33 acts as a pro-tumor factor, while epithelium-derived IL-33 acts as an antitumor factor. This study helps us understand the role of IL-33 in HNSCC and provides some therapeutic implications involving the targeting of this cytokine.

## Data Availability Statement

Publicly available data sets were analyzed in this study. These data can be found here: https://www.cbioportal.org/.

## Ethics Statement

Ethical review and approval was not required for the study on human participants in accordance with the local legislation and institutional requirements. Written informed consent for participation was not required for this study in accordance with the national legislation and the institutional requirements.

## Author Contributions

Conceptualization and design of the study: LP and W-PW. Data collection: LP, WS, and LC. Data analyses: LP, WS, and LC. Data interpretation: all authors. Manuscript writing and reviewing: all authors. All authors contributed to the article and approved the submitted version.

## Funding

This study was supported by the National Natural Science Foundation of China (NSFC) grants No. 81870696, No. 81670902, No. 81470674, and No. 81972527, Guangdong Natural Science Foundation of China grant No. 2018B030312008, and Guangzhou Science and Technology Project of China grants No. 201704020098 and No. 201605030003.

## Conflict of Interest

The authors declare that the research was conducted in the absence of any commercial or financial relationships that could be construed as a potential conflict of interest.

## Supplementary Material

Supplementary Figure 1 The IL-33 expression level and tumor immune microenvironment in laryngeal squamous cell carcinoma. **(A)** IL-33 expression level and immune cell abundances represented by xCell scores; **(B)** IL-33 expression level and molecular markers for tumor immunity. Boxplots represent values within the interquartile range (IQR) (boxes) and 1.5 × IQR (whiskers). Outliers are plotted as values > 1.5 × IQR (circles). P-values were calculated by the Mann-Whitney U test. The Benjamini-Hochberg method was used for multiple testing correction in **(A)**, and p-values with false discovery rates < 0.05 are shown in red. CYT, cytolytic activity.

Supplementary Figure 2 The IL-33 expression level and tumor immune microenvironment in oropharyngeal squamous cell carcinoma. **(A)** IL-33 expression level and immune cell abundances represented by xCell scores; **(B)** IL-33 expression level and molecular markers for tumor immunity. Boxplots represent values within the interquartile range (IQR) (boxes) and 1.5 × IQR (whiskers). Outliers are plotted as values > 1.5 × IQR (circles). P-values were calculated by the Mann-Whitney U test. The Benjamini-Hochberg method was used for multiple testing correction in (A), and p-values with false discovery rates < 0.05 are shown in red. CYT, cytolytic activity.

Supplementary Figure 3 The IL-33 expression level and tumor immune microenvironment in oral cavity squamous cell carcinoma. **(A)** IL-33 expression level and immune cell abundances represented by xCell scores; **(B)** IL-33 expression level and molecular markers for tumor immunity. Boxplots represent values within the interquartile range (IQR) (boxes) and 1.5 × IQR (whiskers). Outliers are plotted as values > 1.5 × IQR (circles). P-values were calculated by the Mann-Whitney U test. The Benjamini-Hochberg method was used for multiple testing correction in **(A)**, and p-values with false discovery rates < 0.05 are shown in red. CYT, cytolytic activity.
