# Supplementary figures and images for: The Role of Interleukin-33 in Head and Neck Squamous Cell Carcinoma Is Determined by Its Cellular Sources in the Tumor Microenvironment
Source: Front Oncol. 2021 Feb 9;10:588454. doi: 10.3389/fonc.2020.588454 (PMC7902021; doi:10.3389/fonc.2020.588454)

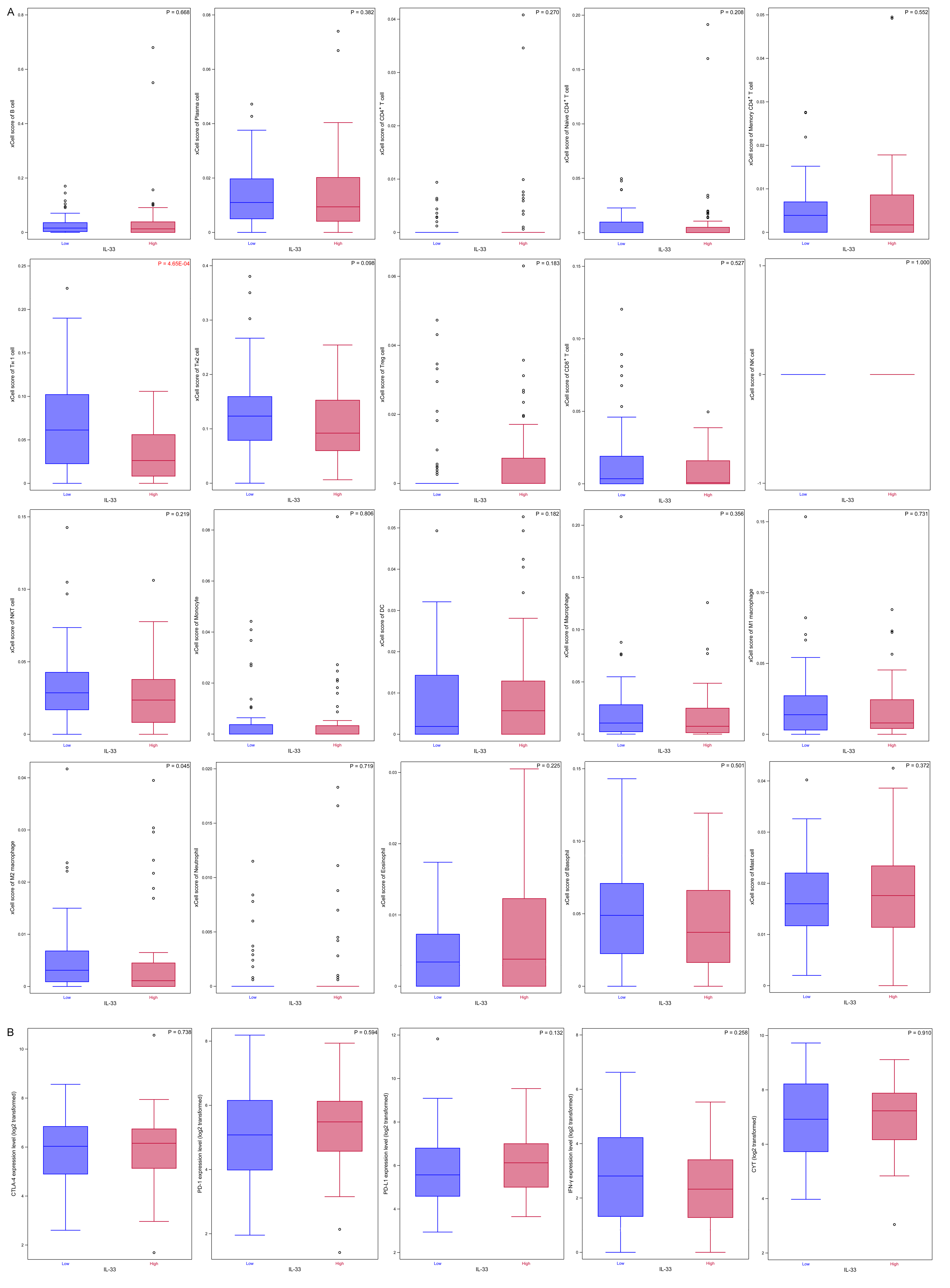

Supplement: Supplementary Figure 1 — The IL-33 expression level and tumor immune microenvironment in laryngeal squamous cell carcinoma. (A) IL-33 expression level and immune cell abundances represented by xCell scores; (B) IL-33 expression level and molecular markers for tumor immunity. Boxplots represent values within the interquartile range (IQR) (boxes) and 1.5 × IQR (whiskers). Outliers are plotted as values > 1.5 × IQR (circles). P-values were calculated by the Mann-Whitney U test. The Benjamini-Hochberg method was used for multiple testing correction in (A), and p-values with false discovery rates < 0.05 are shown in red. CYT, cytolytic activity. [file Image_1.tif]

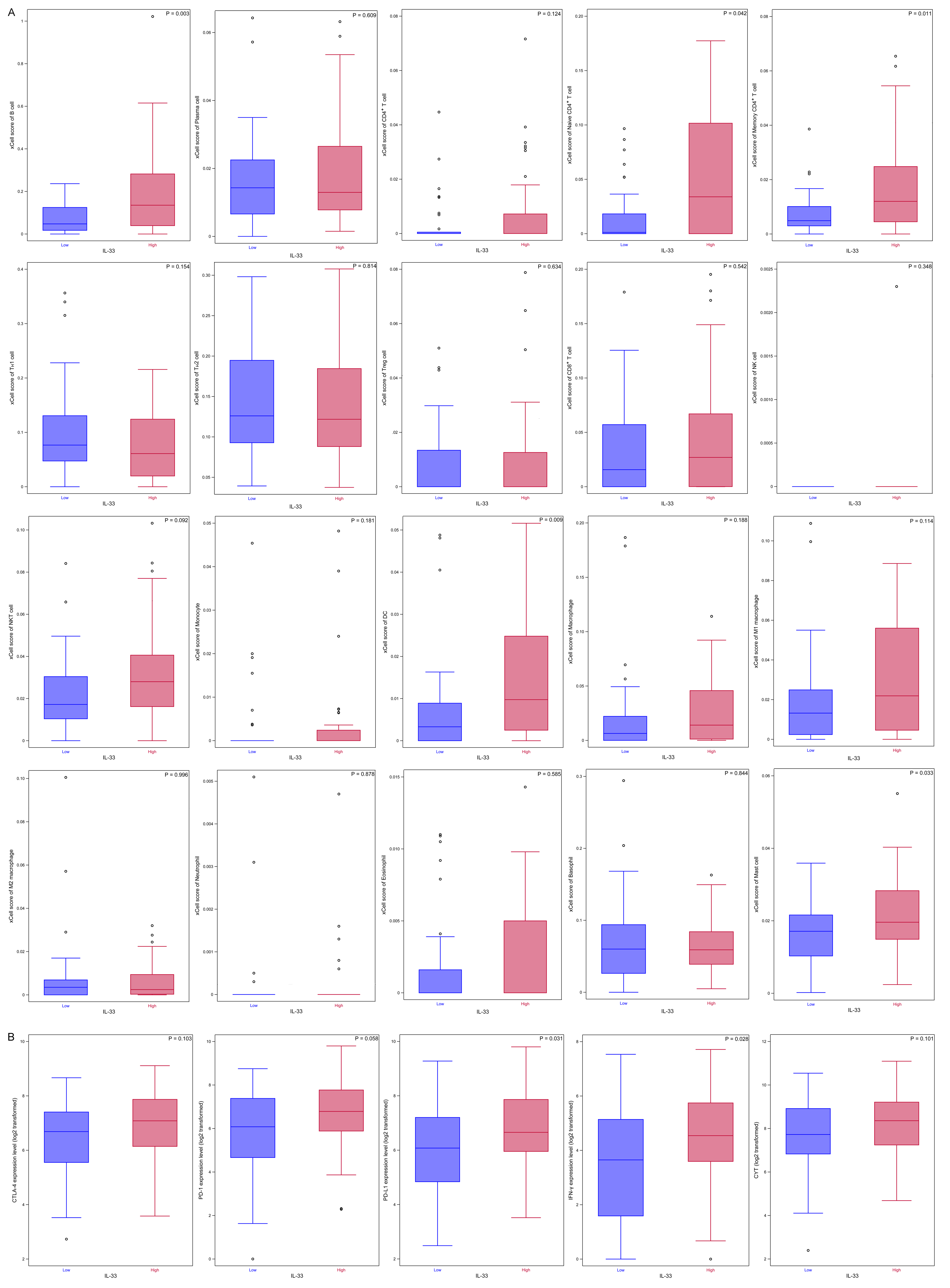

Supplement: Supplementary Figure 2 — The IL-33 expression level and tumor immune microenvironment in oropharyngeal squamous cell carcinoma. (A) IL-33 expression level and immune cell abundances represented by xCell scores; (B) IL-33 expression level and molecular markers for tumor immunity. Boxplots represent values within the interquartile range (IQR) (boxes) and 1.5 × IQR (whiskers). Outliers are plotted as values > 1.5 × IQR (circles). P-values were calculated by the Mann-Whitney U test. The Benjamini-Hochberg method was used for multiple testing correction in (A), and p-values with false discovery rates < 0.05 are shown in red. CYT, cytolytic activity. [file Image_2.tif]

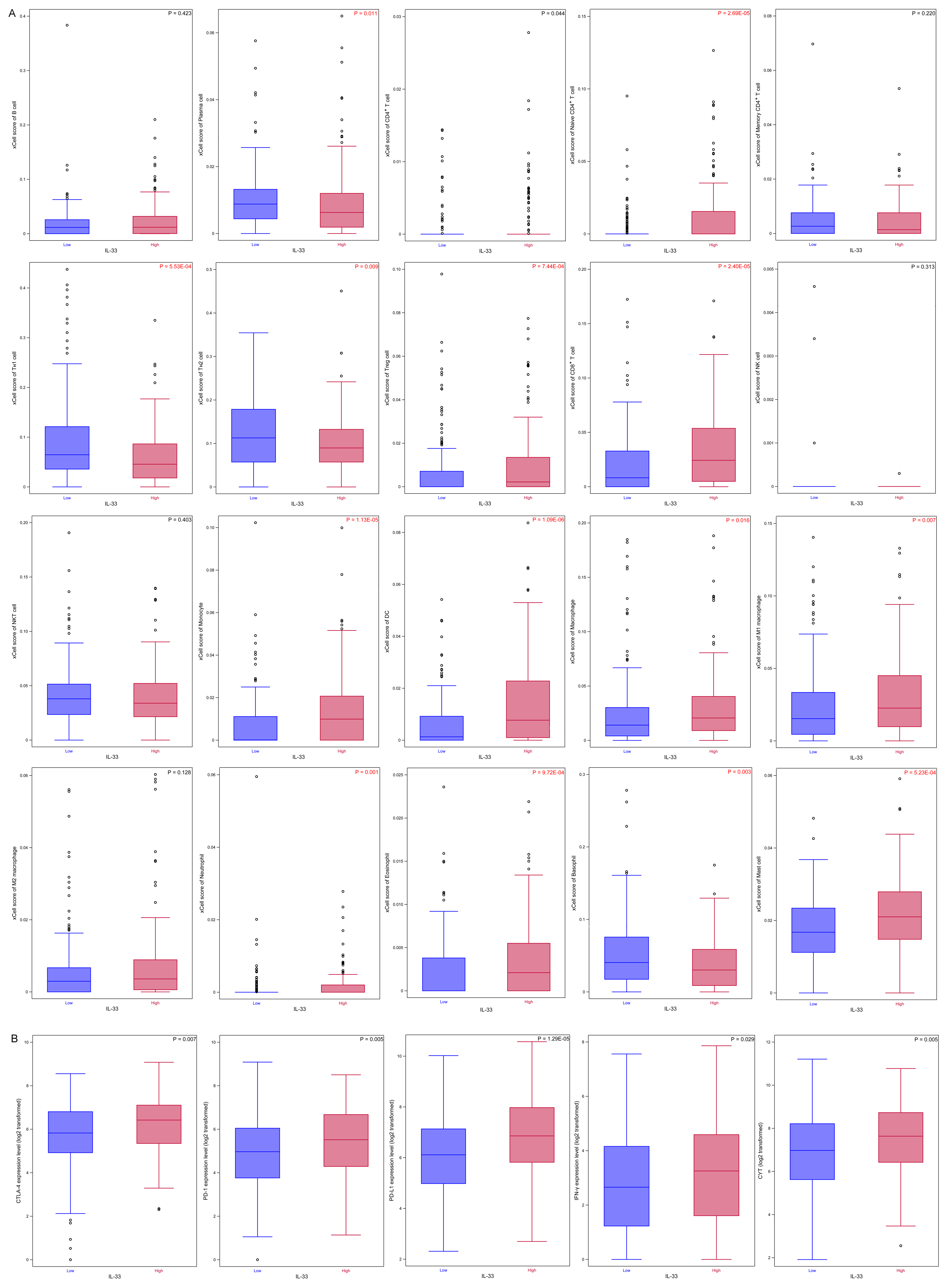

Supplement: Supplementary Figure 3 — The IL-33 expression level and tumor immune microenvironment in oral cavity squamous cell carcinoma. (A) IL-33 expression level and immune cell abundances represented by xCell scores; (B) IL-33 expression level and molecular markers for tumor immunity. Boxplots represent values within the interquartile range (IQR) (boxes) and 1.5 × IQR (whiskers). Outliers are plotted as values > 1.5 × IQR (circles). P-values were calculated by the Mann-Whitney U test. The Benjamini-Hochberg method was used for multiple testing correction in (A), and p-values with false discovery rates < 0.05 are shown in red. CYT, cytolytic activity. [file Image_3.tif]
