# Supplementary material for: The Role of Interleukin-33 in Head and Neck Squamous Cell Carcinoma Is Determined by Its Cellular Sources in the Tumor Microenvironment
Source: Front Oncol. 2021 Feb 9;10:588454. doi: 10.3389/fonc.2020.588454 (PMC7902021; doi:10.3389/fonc.2020.588454)
Supplement: Supplementary file 4 [file Table_1.docx]

**Supplementary Table1. The expression level of IL-33 in subgroups.**

|  | **IL-33 expression level (log2 transformed)** | **p-value^*^** |
| --- | --- | --- |
| **Age** |  | 0.136 |
| ≤ 60y | 6.54 (5.24–8.25) |  |
| > 60y | 7.04 (5.49–8.43) |  |
| **Gender** |  | 0.002 |
| Female | 7.61 (5.82–8.95) |  |
| Male | 6.57 (5.22–8.09) |  |
| **Smoking** |  | 0.103 |
| No | 7.23 (5.71–8.64) |  |
| Yes | 7.42 (5.43–8.79) |  |
| **Drinking** |  | 0.698 |
| No | 6.96 (5.41–8.33) |  |
| Yes | 6.70 (5.38–8.29) |  |
| **HPV status** |  | 0.351 |
| Negative | 6.78 (5.20–8.23) |  |
| Positive | 7.02 (5.65–8.45) |  |
| **Tumor site** |  | 0.535 |
| Hypopharynx | 7.66 (3.25–10.79) |  |
| Larynx | 6.57 (5.40–7.90) |  |
| Oropharynx | 7.41 (5.59–8.47) |  |
| Oral cavity | 6.78 (5.22–8.41) |  |
| **Grade** |  | 0.382 |
| 1 | 7.20 (5.40–8.58) |  |
| 2 | 6.71 (5.20–8.17) |  |
| 3 | 6.86 (5.39–8.34) |  |
| 4 | 7.91 (6.06–9.59) |  |
| **T stage** |  | < 0.001 |
| T1 | 8.00 (6.34–8.94) |  |
| T2 | 7.13 (5.81–8.42) |  |
| T3 | 6.70 (5.49–8.03) |  |
| T4 | 6.24 (4.67–8.01) |  |
| **N stage** |  | 0.386 |
| N0 | 7.04 (5.20–8.54) |  |
| N1 | 6.84 (5.35–7.88) |  |
| N2 | 6.45 (5.20–8.00) |  |
| N3 | 6.33 (3.98–8.40) |  |
| **Overall stage** |  | < 0.001 |
| I | 8.51 (7.06–9.48) |  |
| II | 7.13 (5.87–8.54) |  |
| III | 7.00 (5.50–8.10) |  |
| IV | 6.39 (5.06–7.99) |  |

Data presented as median (interquartile range).

^*^Mann-Whitney U-test or Kruskal-Wallis test.
