# Supplementary material for: The Role of Interleukin-33 in Head and Neck Squamous Cell Carcinoma Is Determined by Its Cellular Sources in the Tumor Microenvironment
Source: Front Oncol. 2021 Feb 9;10:588454. doi: 10.3389/fonc.2020.588454 (PMC7902021; doi:10.3389/fonc.2020.588454)
Supplement: Supplementary file 5 [file Table_2.docx]

**Supplementary Table S2. The immune cell abundances and expression level of molecular markers for tumour immunity between the high IL-33 group and the low IL-33 group.**

|  | **The whole cohort** | | |
| --- | --- | --- | --- |
|  | Low IL-33 | High IL-33 | p-value^*^ |
|  | N = 260 | N = 260 |  |
| **Immune cells (xCell scores)** | | | |
| B cell | 0.014 (0–0.037) | 0.017 (0.001–0.053) | 0.107 |
| Plasma cell | 0.010 (0.005–0.017) | 0.008 (0.003–0.015) | 0.043 |
| CD4+ T cell | 0 (0–0) | 0 (0–0) | **0.006** |
| CD4+ naïve T cell | 0 (0–0.003) | 0 (0–0.018) | **< 0.001** |
| CD4+ memory T cell | 0.004 (0–0.008) | 0.003 (0.008–0.009) | 0.971 |
| TH1 cell | 0.065 (0.034–0.114) | 0.043 (0.016–0.080) | **< 0.001** |
| TH2 cell | 0.117 (0.070–0.173) | 0.098 (0.061–0.147) | **0.005** |
| Treg cell | 0 (0–0.006) | 0 (0–0.013) | **< 0.001** |
| CD8+ T cell | 0.007 (0–0.030) | 0.017 (0-0.042) | **< 0.001** |
| Natural killer cell | 0 (0-0) | 0 (0-0) | 0.648 |
| Natural killer T cell | 0.033 (0.018-0.048) | 0.030 (0.018–0.049) | 0.520 |
| Monocyte | 0 (0–0.007) | 0.003 (0–0.017) | **< 0.001** |
| Dendritic cell | 0.002 (0–0.010) | 0.007 (0.001–0.022) | **< 0.001** |
| Macrophage | 0.012 (0.002–0.029) | 0.017 (0.004–0.038) | **0.030** |
| Macrophage M1 | 0.015 (0.003–0.030) | 0.020 (0.006–0.043) | **0.007** |
| Macrophage M2 | 0.003 (0–0.007) | 0.003 (0–0.009) | 0.743 |
| Neutrophil | 0 (0-0) | 0 (0–0.001) | **0.005** |
| Eosinophil | 0 (0-0.004) | 0.002 (0–0.007) | **< 0.001** |
| Basophil | 0.047 (0.021-0.081) | 0.036 (0.012–0.065) | **0.010** |
| Mast cell | 0.017 (0.011-0.023) | 0.020 (0.014–0.027) | **< 0.001** |
| **Molecular markers expression level (log2 transformed)** | | | |
| CTLA-4 | 5.96 (4.98–6.96) | 6.52 (5.46–7.18) | 0.001 |
| PD-1 | 5.05 (3.94–6.25) | 5.64 (4.49–6.76) | < 0.001 |
| PD-L1 | 6.05 (4.80–7.10) | 6.61 (5.60–7.86) | < 0.001 |
| IFN-γ | 2.76 (1.27–4.24) | 3.24 (1.62–4.59) | 0.022 |
| Cytolytic activity | 7.10 (5.82–8.33) | 7.63 (6.49–8.56) | 0.002 |

Data presented as median (interquartile range).

^*^Mann-Whitney U-test. The Benjamini-Hochberg method was used for multiple testing correction in comparisons of the immune cell abundances, and p-values with false discovery rates < 0.05 are shown in bold.
